# Endoscopic self-expandable metal stent versus endoscopy vacuum therapy for traumatic esophageal perforations: a retrospective cohort study

**DOI:** 10.1007/s00464-024-10755-5

**Published:** 2024-03-06

**Authors:** Alessandrino Terceiro de Oliveira, Márcio Alencar Barreira, José Wilson da Cunha Parente Júnior, José Ruver Lima Herculano Junior, Jeany Borges e Silva Ribeiro, Orleancio Gomes Ripardo de Azevedo, Paulo Roberto Cavalcante de Vasconcelos

**Affiliations:** 1 Department of Digestive Endoscopy, Dr. José Frota Institute, Fortaleza, CE Brazil; 2 Department of Surgery, Federal University of Ceara, Fortaleza, CE Brazil; 3 Department of Digestive Endoscopy, General Hospital of Fortaleza, Fortaleza, CE Brazil; 4 Department of Surgery, Dr. José Frota Institute, Fortaleza, CE Brazil; 5 Department of Digestive Endoscopy, Federal University of Piaui, Teresina, PI Brazil; 6 Department of Surgery, Federal University of Ceara, 1608 N. Prof Costa Mendes St, 3rd Floor, Fortaleza, CE 60416-200 Brazil

**Keywords:** Esophageal perforation, Esophageal diseases, Endoscopic self-expandable metal stent, Endoscopy vacuum therapy, Cohort studies

## Abstract

**Background:**

Traumatic esophageal perforations (TEP) are a grave medical condition and require immediate intervention. Techniques such as Esophageal Self-Expandable Metal Stent (E-SEMS) and Endoscopic Vacuum Therapy (EVT) show promise in reducing tissue damage and controlling esophageal leakage. The present study aims to compare the application of EVT to E-SEMS placement in TEP.

**Methods:**

Retrospective cohort study valuated 30 patients with TEP. The E-SEMS and EVT groups were assessed for time of hospitalization, treatment duration, costs, and clinical outcome.

**Results:**

Patients treated with EVT (24.4 ± 13.2) demonstrated significantly shorter treatment duration (*p* < 0.005) compared to the group treated with E-SEMS (45.8 ± 12.9) and patients submitted to E-SEMS demonstrated a significant reduction (*p* = 0.02) in the time of hospitalization compared to the EVT (34 ± 2 vs 82 ± 5 days). Both groups demonstrated a satisfactory discharge rate (E-SEMS 93.7% vs EVT 71.4%) but did not show statistically significant difference (*p* = 0.3155). E-SEMS treatment had a lower mean cost than EVT (*p* < 0.05). Descriptive statistics were utilized, arranged in table form, where frequencies, percentages, mean, median, and standard deviation of the study variables were calculated and counted. The Fisher's Exact Test was used to evaluate the relationship between two categorical variables. To evaluate differences between means and central points, the parametric t-test was utilized. Comparisons with p value up to 0.05 were considered significant.

**Conclusion:**

E-SEMS showed a shorter time of hospitalization, but a longer duration of treatment compared to EVT. The placement of E-SEMS and EVT had the same clinical outcome. Treatment with E-SEMS had a lower cost compared with EVT.

Esophageal injury is a grave medical condition, with mortality rates between 10% and 25% when treatment is started within the first 24 h, escalating to 40–60% if the treatment is delayed [1]. Traumatic esophageal perforations (TEP) due to foreign bodies are rare but carry potentially devastating complications [2]. Penetrating mechanisms caused by violence, such as gunshot and stab wounds, require immediate intervention because of their potential to cause indiscernible tissue damage [3].

Such injuries can rapidly escalate into life-threatening situations due to the potential of mediastinal contamination following esophageal leakage, leading to sepsis [4]. Gunshot wounds are of particular concern due to the risk of ischemia-induced necrosis. Conversely, stab wounds, although usually inflicting less tissue damage, present a significant threat by potentially harming crucial blood vessels [5]. Foreign bodies in the esophagus, an underappreciated cause of injury, can lead to complications such as mucosal abrasion, perforation, and mediastinitis [6].

Effective management of TEP necessitates immediate patient stabilization, employing large-bore intravenous catheters, isotonic crystalloids, and broad-spectrum antibiotics [7]. While surgical intervention remains a primary approach, there is growing interest in less invasive alternatives. Techniques like Esophageal Self-Expandable Metal Stent (E-SEMS) and Endoscopic Vacuum Therapy (EVT) show promise in reducing tissue damage and controlling esophageal leakage [8, 9]. Thus, these approaches present potential advancements in the management of esophageal injuries [10].

The use of endoscopy therapies in TEP can reduce time of hospitalization and, consequently, costs with a favorable clinical outcome. The present study aims to compare the application of EVT to E-SEMS placement in TEP.

## Materials and methods

Retrospective cohort study was started after approved by the Institutional Research Ethics Board (number 5.704.940) and Brazil platform system (Approval with CAAE number 61492122.0.0000.5047). The study was conducted in compliance with the Declaration of Helsinki and data were collected from medical records.

### Setting and study population

The research was carried out in emergency department of the Dr. José Frota institute, located in the city of Fortaleza. The largest trauma center in Northeast Brazil.

Patients with associated extra thoracic injuries were not selected for the study. An initial selection of 44 patients with traumatic esophageal injuries took place between May 2020 and May 2022. However, 14 patients were excluded from the study. Exclusion criteria for the study were the association of foreign body perforations with malignant neoplasms of the esophagus (*n* = 8), death before proposed treatment due to numerous vascular lesions (*n* = 2), multiple chest injuries and hemodynamic instability that were treated by thoracotomy (*n* = 4). 30 patients were distributed into the groups that received the EVT (*n* = 14) or E-SEMS (*n* = 16) intervention before completing 24 h of injury. No patient was lost to follow-up.

### Assessments and outcomes

The primary outcomes were time of hospitalization, treatment duration, costs, and clinical outcome. The secondary outcomes were sex, age, location, and cause of the esophageal perforation.

The location of esophageal lesions considered the distance from the upper dental arch. The patients were categorized based on the cause of their injuries: gunshot wounds, stab wounds, or injuries from foreign bodies. The time of hospitalization, and treatment duration were recorded in days. The length of stay accounted for the period that the patient remained in the hospital during the first hospitalization. Treatment ended when the injury healed, food was allowed, endoscopic therapies were suspended, and the patient was discharged from the hospital. The cost analysis considered daily hospital stays and materials for endoscopic therapies. The clinical outcome was divided into discharge or death. Discharged patients had complete healing of the esophageal perforation.

### Interventions

The patients selected for the study underwent upper gastrointestinal endoscopy with minimal air insufflation after orotracheal intubation. All endoscopic procedures were performed by an endoscopist with expertise in endoscopic techniques using a Pentax EPK-i video endoscope and a regular Pentax EG-2790i video gastroscope.

### EVT

Initially, debridement of the lesion was performed using a regular biopsy forceps (FB-21K-1, Olympus, Japan) and the wound was washed with 2% hydrogen peroxide solution.

The system used in the EVT was a low-cost system [11] adapted by Dr. Flaubert Sena de Medeiros using a nasogastric tube (Levin 16 French, Insung Medical, Seoul, Korea) covered with sterile gauze and laparoscopic plastic inserted into one nostril and then gripped in the oral cavity using a forceps (FG-42L-1, Olympus, Japan), and directed to the esophageal lesion site. In addition, based on the size of the lesion, two types of EVT were used: a short system (< 5 cm) used intracavitary or a long system (> 5 cm) used intraluminally.

After the placement of the vacuum therapy system, the nasogastric tube was connected and adjusted to a continuous negative pressure between 80 and 125 mmHg. The system was changed approximately twice a week until complete healing of the lesion. At the start of EVT placement, patients maintained two nasal tubes: a vacuum therapy tube in one nostril and a nasoenteral tube for enteral feeding in another nostril. After complete healing of the fistula, patients returned to oral feeding.

### E-SEMS

The placement of the self-expandable and fully covered metal prosthesis took place under radioscopic control in the operating room. The types of prostheses used were: Hanaro (Tokyo, Japan) and WallFlex (Massachusetts, USA) with a diameter of 23 mm and variable length depending on the location of the perforation. All prostheses used were not fixed with metal clips or endoscopic suture and as a rule were left at least 4 cm below the cricopharyngeus to avoid the sensation of a foreign body. It should be noted that in patients with perforations up to 4 cm below the cricopharyngeus, vacuum therapy was mandatory considering the discomfort caused by the self-expanding prosthesis in these cases.

The patient was instructed to maintain about 48 h of bed rest in the dorsal decubitus position to prevent prosthesis migration. After this period, a chest CT scan with oral non-ionic contrast was performed to check the position of the prosthesis. If properly positioned and the patient clinically stable, the hospital discharge was carried out. If prosthesis migration occurred within 48 h, the patient then underwent a new endoscopic procedure for the correct repositioning of it with the help of a foreign body forceps. The patient was advised to maintain a liquid diet until the esophageal stent was removed. About 6 weeks after prosthesis placement, patients returned to the hospital's gastrointestinal endoscopy department for its removal via endoscopy, with lesion healing being assessed.

### Statistical analysis

The data was tabulated using Microsoft Excel 2016 software. Descriptive statistics were utilized, arranged in table form, where frequencies, percentages, mean, median, and standard deviation of the study variables were calculated and counted. Following initial evaluations of the data, statistical methods were applied to verify associations and correlations between variables. The Fisher's Exact Test was used to evaluate the relationship between two categorical variables. To evaluate differences between means and central points, the parametric t-test was utilized. Comparisons with p value up to 0.05 were considered significant.

## Results

Table 1 details the variables analyzed (sex, age, cause and location of the perforation, time of hospitalization, treatment duration, and clinical outcome) in all patients. Data were separated by type of endoscopic intervention (E-SEMS or EVT). Of the 16 patients treated with an E-SEMS, 11(68.75%) were men and 5 (31.25%) were women. In 14 patients treated by EVT, 9 (62.28%) were men and 5 (35.72%) were women. A total of 30 patients, 20 men and 10 women. The mean age of patients in the E-SEMS was 48 ± 17 years and EVT was 44 ± 27 years. The patients' ages ranged from 6 to 81 years.

**Table 1:** Study population classified by type of endoscopic intervention in TEP

Sex	Age	Cause	Upper dental arch*	Time of hospitalization, days	Treatment duration, days	Clinical outcome
E-SEMS						
M	36	Gunshot wound	22	25	42	Discharge
M	18	Stab	22	41	32	Discharge
M	46	Foreign body	24	28	53	Discharge
M	45	Foreign body	30	56	62	Discharge
F	67	Foreign body	24	92	48	Discharge
M	45	Foreign body	33	34	67	Discharge
F	78	Foreign body	31	22	58	Discharge
M	65	Gunshot wound	26	78	45	Discharge
M	38	Foreign body	33	36	54	Discharge
F	59	Foreign body	31	35	61	Discharge
M	23	Gunshot wound	25	22	44	Discharge
M	31	Gunshot wound	29	22	32	Discharge
M	66	Foreign body	30	13	24	Discharge
M	62	Foreign body	32	29	26	Death
F	20	Foreign body	24	6	42	Discharge
F	45	Foreign body	35	8	44	Discharge
EVT						
M	75	Foreign body	22	52	15	Discharge
F	81	Foreign body	25	96	22	Discharge
F	18	Gunshot wound	27	142	41	Discharge
M	20	Gunshot wound	32	103	15	Discharge
M	41	Gunshot wound	33	221	62	Death
M	6	Foreign body	20	97	22	Discharge
M	67	Foreign body	31	62	14	Discharge
F	72	Foreign body	31	36	15	Discharge
M	22	Gunshot wound	30	52	20	Discharge
F	27	Stab	18	22	14	Discharge
M	74	Foreign body	30	68	24	Death
M	62	Foreign body	32	35	21	Death
M	18	Gunshot wound	24	78	25	Discharge
F	39	Foreign body	22	96	32	Death

*M* male, *F* female

*Refers to the distance from the perforation in the esophagus to the dental arch

A 6-year-old child presented with a perforation in the esophagus (20 cm from the upper dental arch) due to a foreign body. The unavailability of pediatric prostheses meant that the patient was included in the EVT group, increasing the number of patients in this group.

Table 2 summarized the dividing patients by causes of esophageal perforation (foreign body, gunshot, and stab wounds). The group treated with E-SEMS was distributed into (11/16) (4/16) and (1/16), respectively. For patients treated with EVT, the distribution was (8/14), (5/14), (1/14), respectively. In general, the causes that led patients to the hospital ranged from foreign body 63.3% (19/30), followed by gunshot wounds 30% (9/30) and stab wounds 6.7% (2/30).

**Table 2:** Patient distribution according to the trauma injuries

Trauma injuries	E-SEMS	EVT
Foreign body	11/16 (68, 75%)	08/14 (57, 14%)
Gunshot wounds	04/16 (18, 75%)	05/14 (35, 71%)
Stab wounds	01/16 (6, 25%)	01/14 (7, 14%)

**Fig. 1:**
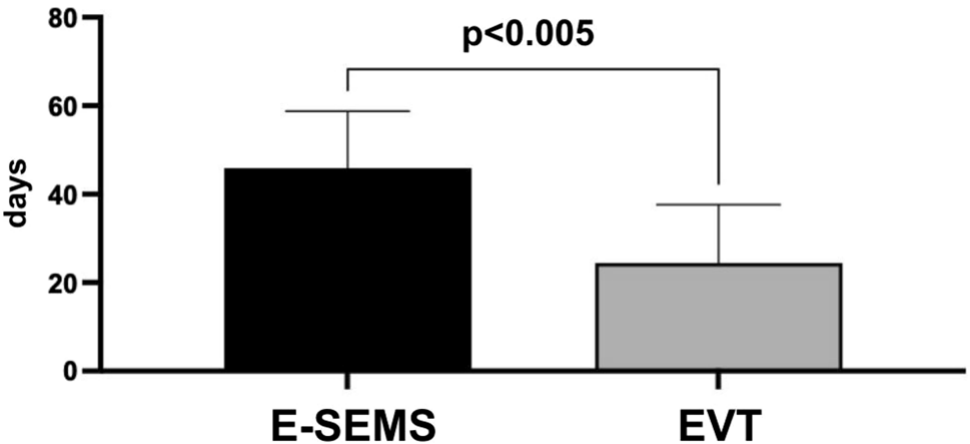
Treatment duration of patients with TEP treated with E-SEMS and EVT

Figure 1 shows that patients treated with EVT (24.42 ± 13.21) demonstrated significantly shorter treatment duration compared to the group treated with E-SEMS (45.87 ± 12.90). The value of *p* < 0.005 shows that there is a statistically significant difference.

**Fig. 2:**
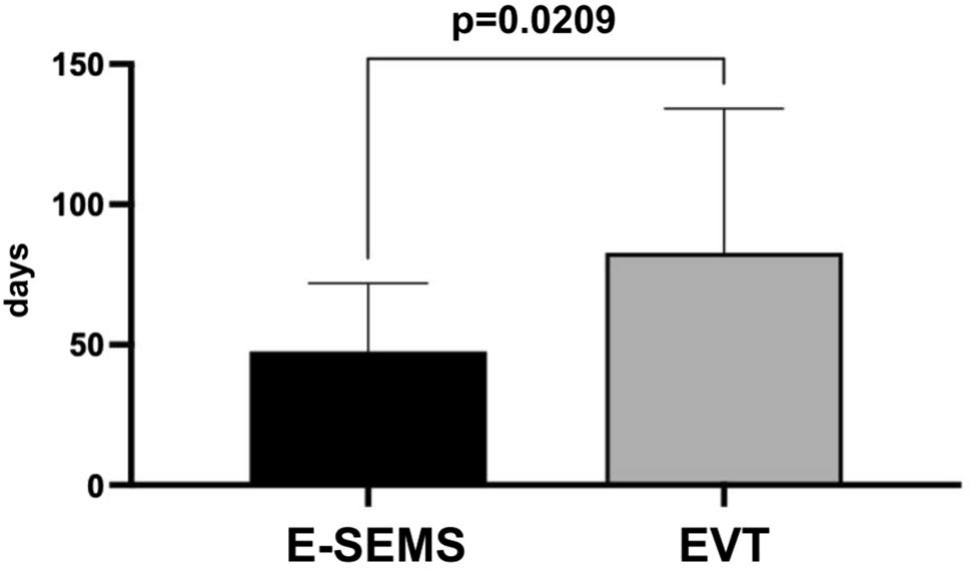
Time of hospitalization in patients with TEP treated with E-SEMS and EVT

On the other hand, patients submitted to E-SEMS demonstrated a significant reduction (*p* = 0.0209) in the time of hospitalization compared to the EVT (34 ± 23 vs 82 ± 51) (Fig. 2). Prosthesis migration occurred in three patients, one of these patients underwent two procedures to exchange the prosthesis, which led to a total of 92 days of treatment.

In clinical outcomes, both groups demonstrated a satisfactory discharge rate (E-SEMS 93.7% vs EVT 71.4%). In the EVT group, there were 28.6% (4/14) deaths compared to 6.25% (1/16) that were treated with the E-SEMS. However, the groups did not show statistically significant difference (*p* = 0.3155). There was a total of five deaths. The four deaths in the EVT group, the cause was sepsis after the patients developed mediastinitis. The one death in the E-SEMS group, the cause was associated with heart failure unrelated to the study.

The mean cost for the two techniques was evaluated and E-SEMS treatment had a lower mean cost than EVT. The data were analyzed using the application of the parametric *t* Test and the non-parametric Mann–Whitney test. Both tests were significant (*p* < 0.05) (Table 3).

**Table 3:** Cost comparison between E-SEMS and EVT treatments

Treatment	Cost*	t-test	Mann–Whitney
E-SEMS	16,626.60ª (19,266.08 ± 10,925.00)	0.013	0.005
EVT	33,8878.60ª (38,453.17 ± 23,840.40)		

^a^Median, (mean ± SD)

## Discussion

The esophageal lesions treatment, with a mortality rate of around 20%, remains a clinical challenge [12]. However, recently, less invasive techniques, such as endoscopic procedures, have become more prevalent, resulting in decreased mortality [13]. One of the most used endoscopic treatments is E-SEMS, which has been shown to be effective in reducing the mortality rate compared to surgery [14].

In contrast, EVT gained prominence for offering advantages such as lower costs, shorter Intensive Care Unit stay, and shorter Length of Hospital Stay compared to surgical interventions [15]. In this study, it was noted that patients undergoing EVT spent less time in treatment when compared to E-SEMS, although the duration of hospitalization was longer for patients in the EVT group, due to the need for outpatient removal of the E-SEMS after hospital discharge.

Cost analysis demonstrated that treatment with E-SEMS was significantly cheaper for brazilian unified health system compared to EVT, mainly due to the shorter hospital stay in the E-SEMS group. However, some studies did not show significant differences in length of stay between the two methods [16].

The effectiveness of the two treatment methods was also compared. This study, in line with other previous studies [17, 18], found no significant differences in success rates between groups. However, other studies suggest greater efficacy for EVT [19, 20].

Regarding mortality, although this study showed a higher number of deaths in the EVT group (28.6%) compared to the E-SEMS group (6.25%), this difference was not statistically significant. The main cause of death in the EVT group was septicemia, probably due to the longer length of hospital stay [18].

As for complications, EVT did not present complications related to the technique, with the greatest morbidity factor being the longer length of hospital stay, which increases the risk of infection. On the other hand, the major complication of E-SEMS was stent migration, which occurred in 18% of patients [21].

Both E-SEMS and EVT methods have been shown to be effective in treating esophageal perforations, each has its own advantages and disadvantages. The choice of method should be based on an individual assessment of each patient, considering the clinical practice, the patient’s preferences, and the cost of treatment for the health system.

It should be noted that, compared to several studies analyzed in the literature, in the present study all patients treated with E-SEMS were discharged early after tomographic verification of the correct positioning of the stent. This conduct was probably relevant in reducing the group's morbidity and mortality, as it spared patients from comorbidities and the prevalence of infections associated with prolonged hospital stays.

In conclusion, the placement of E-SEMS and EVT had the same clinical outcome. The group of patients treated with endoscopic implantation of the E-SEMS showed a shorter hospital stay with a longer duration of out-of-hospital treatment. The comparative analysis of the costs associated with the placement of the E-SEMS and EVT procedures demonstrates a significantly higher expense for EVT.
